# Supplementary material for: Assessment of effectiveness of DAMaN: A malaria intervention program initiated by Government of Odisha, India
Source: PLoS One. 2020 Sep 8;15(9):e0238323. doi: 10.1371/journal.pone.0238323 (PMC7478908; doi:10.1371/journal.pone.0238323)
Supplement: S1 File — (PDF) [file pone.0238323.s001.pdf]

PART I

HOUSEHOLD DETAILS

DISTRICT NAME ________________________ DISTRICT NO [ ]<br>BLOCK NAME ___________________________ BLOCK NO [ ]<br>SUB CENTRE NAME ___________________ SUB CENTRE [ ]<br>HOUSEHOLD NO [ ] [ ]<br>NAME OF HOUSEHOLD HEAD ______	
GPS Coordinates LAT [ ].[ ] LONG [ ].[ ]	GPS coordinates in units of decimal degree
TOTAL PERSONS IN THE HOUSEHOLD [ ]	
CASTE [ ]<br>NAME OF THE TRIBE (If caste is ST) __________________	GENERAL=1<br>OBC=2<br>SC=3<br>ST=4<br>If 'CASTE' is ST, then write the name of the tribe

SECTION 1

INFORMATION ABOUT HOUSEHOLD MEMBERS

Individual Unique I.D	Name of the House Hold Members	Relationship with HH	Gender	Age ***	Education ****	Previous DAMaN Results					Hb (g/dl) *	Anthropometry**			RDT	Microscopy	PCR
						Test pos	Test neg	Tested, Doesn't Remember	Not Tested	Verified by patient card Yes-1, No-0 Don't know-98		Ht (cm)	Wt (Kg)	MUAC (cm)			

*Hemoglobin(Hb) will be calculated for Under-5 children and Mother only

**Anthropometry for Under-5 Children only

***Record in completed months

**** Education in completed years

SECTION 2

SOCIO ECONOMIC CHARACTERISTICS

No.	QUESTIONS AND FILTERS	CODING CATEGORIES		INSTRUCTION
1.	Do you or your family own this dwelling?	Yes	1	
		No	0	
2.	What is the main source of drinking water for members of your household?	Piped water 1<br>Tube well or borehole……… 2<br>**Dug well……….. 3**<br>**Water from spring………. 4**<br>Tanker truck………………5<br>Surface water (river/dam/ lake/pond/stream/canal/ Irrigation channel)……………....6		
3.	What does this household usually do to make the water safer to drink? Anything else?	Nothing……………….…………..1<br>Boil ………………………………..2<br>Use alum…………………………3<br>Add bleach/chlorinetablets……...4<br>Strain through a cloth……………5<br>Use water filter (ceramic/ Sand/composite/etc.)……………6<br>Use electronic purifier……………7		.<br>. . . . .<br>X
4.	What kind of toilet facility does your household use?	FLUSH TOILET ………………1<br>PIT TOILET LATRINE……….2<br>NO FACILITY`……………….3		
5.	Does your household have / own:	YES	NO	
	Electricity?	1	0	
	A radio?	1	0	
	A television?	1	0	
	A mobile telephone?	1	0	
	A refrigerator?	1	0	
	Bank or post-office account?	1	0	
	Bicycle?	1	0	
	Scooter/motorcycle?	1	0	
	Car/truck/tractor?	1	0	
	Separate room for cooking?	1	0	
	Livestock (goats, cow, buffalo, bull, sheep, pig)?	1	0	
	Separate room for livestock	1	0	

6.	WHAT TYPE OF FUEL DOES YOUR HOUSEHOLD **MAINLY** USE FOR COOKING?	1 = Wood<br>2 = Coal, lignite<br>4 = Kerosene<br>5 = Charcoal<br>6 = Animal dung<br>7 = LPG/Natural gas<br>8 = Electricity<br>9 = Straw/shrubs/grass<br>10 = Agricultural crop<br>11 = Biogas<br>98= DON'T KNOW	
7.	**MAIN** MATERIAL OF THE FLOOR.<br><br>RECORD OBSERVATION.	1=NATURAL<br><br>2=BASIC<br><br>3=FINISHED	**NATURAL** =<br>Mud/clay/earth, Sand, Dung<br>**BASIC**=<br>Raw wood planks, Palm, bamboo, Brick, Stone<br>**FINISHED**=<br>Parquet, polished wood, Vinyl, asphalt strips, Ceramic tiles, Cement, Carpet, Polished stone/marble/granite
8.	**MAIN** MATERIAL OF THE WALL.<br><br>RECORD OBSERVATION.	1=NATURAL<br><br>2=BASIC<br><br>3=FINISHED	**NATURAL**=<br>No walls,<br>Cane/palm/trunks, Mud,<br>Grass/reeds/thatch<br><br>**BASIC** = Bamboo with mud, Stone with mud, Plywood, Cardboard, Unburnt brick, Raw/reused wood<br>**FINISHED**=<br>Cement/concrete, Stone with lime/cement, Burnt bricks, Cement blocks, Wood planks/shingles, GI/metal/asbestos sheets
9.	**MAIN** MATERIAL OF THE ROOF.<br><br>RECORD OBSERVATION.	1=NATURAL<br><br>2=BASIC<br><br>3=FINISHED	**NATURA**L=<br>No roof, Thatch/palm leaf<br>Mud, Sod/mud and grass mixture,<br>Plastic/polythene sheeting<br><br>**BASIC**=<br>Rustic mat,<br>Palm/bamboo, Raw wood planks/timber, Unburnt bricks, Loosely packed stone<br><br>**FINISHED**=<br>Metal/GI, Wood, Calamine/cement fiber, Asbestos sheets, RCC/RBC/cement/concrete, Roofing shingles, Tiles, Slate, Burnt brick

10.	TYPE OF WINDOWS<br><br>RECORD OBSERVATION.		YES	NO	
		WINDOWS WITH GLASS	1	0	
		WINDOWS WITH SCREENS	1	0	
		WINDOWS WITH CURTAINS OR SHUTTERS	1	0	
		ANY OTHER VENTILATION	1	0	
11.	HOW MANY ROOMS IN THIS HOUSEHOLD ARE USED FOR SLEEPING?<br>Include only rooms which are usually used for sleeping.	NUMBER OF SLEEPING ROOMS	__ __		
12.	DOES THIS HOUSEHOLD HAVE A BPL CARD?	Yes	1		
		No	0		
13.	IS ANY USUAL MEMBER OF THIS HOUSEHOLD COVERED BY A HEALTH SCHEME OR HEALTH INSURANCE?	Yes	1		
		No	0		

SECTION 3

GENERAL MALARIA KNOWLEDGE / PRACTICES

NO.	QUESTIONS AND FILTERS	CODING CATEGORIES	Instructions data collection	Instructions data entry
14	CAN YOU TELL ME THE MAIN SIGNS OR SYMPTOMS OF MALARIA?<br><br>MULTIPLE RESPONSES POSSIBLE<br>PROBE ONCE (ANYTHING ELSE?)	FEVER……………………………………..<br>CHILLS……………………………………<br>FEELING COLD…………………………..<br>HEADACHE……………………………….<br>NAUSEA AND VOMITING………………<br>DIARRHEA……………………………….<br>DIZZINESS………………………………..<br>LOSS OF APPETITE……………………..<br>BODY ACHE OR JOINT PAIN………….<br>PALE EYES……………………………….<br>BODY WEAKNESS………………………<br>DON'T KNOW…………………………….	number to be given rank(s) as the answer(s) received	There will be 5 variables with all the 14 possible values for each of them.<br><br>The first ranked answer should be entered under the first variable. The second ranked answer will be entered under the second variable…so on. When any variable has NA or Don't Know as an answer all subsequent variables should be NA(empty)
15	IN YOUR OPINION, WHAT CAUSES MALARIA?<br><br>MULTIPLE RESPONSES POSSIBLE<br>PROBE ONCE (ANYTHING ELSE?)	MOSQUITO BITES………………<br>EATING DIRTY FOOD…...<br>DRINKING DIRTY WATER………………..<br>GETTING SOAKED WITH RAIN………….<br>COLD OR CHANGING WEATHER………<br>CONTACT WITH SICK PERSON……..<br>WITCHCRAFT…………………………<br>ENVIRONMENT…………………………<br>DON'T KNOW...	All possible numbers to be given rank as the answer(s) received	There will be 5 rank variables with all the possible values for each of them.<br><br>For rest refer question seven
16	HOW CAN SOMEONE PROTECT THEMSELVES AGAINST MALARIA?<br><br>MULTIPLE RESPONSES POSSIBLE<br>PROBE ONCE (ANYTHING ELSE?)	SLEEP UNDER A MOSQUITO NET……..<br>USE MOSQUITO REPELLANT………......<br>COVERED CLOTHS………………..<br>TAKE PREVENTIVE MEDICATION……...<br>SPRAY HOUSE WITH INSECTICIDE……<br>CUT GRASS BY THE HOUSE……<br>FILL IN PUDDLES (STAGNANT WATER)……………………………………..<br>KEEP HOUSE AREA CLEAN……………<br>BURN LEAVES / CREATE SMOKE………………………<br>DON'T DRINK DIRTY WATER…………..<br>DON'T EAT BAD FOOD…………..……..<br>PUT SCREENS ON THE WINDOWS..…<br>DON'T GET SOAKED WITH RAIN……...<br><br>DON'T KNOW……………………………..	All possible numbers to be given rank as the answer(s) received	There will be 5 rank variables with 15 possible values for each of them.<br><br>For rest refer question seven
17	HAVE YOU EVER HEARD OR SEEN ANY MESSAGES / INFORMATION ABOUT MALARIA?	YES...1<br>NO...0	IF 'NO' SKIP 18,19,20	

18	WHERE DID YOU SEE OR HEAR THESE MESSAGES/INFORMATION?<br>MULTIPLE RESPONSES POSSIBLE<br><br>PROBE ONCE (ANYTHING ELSE?)	GOVERNMENT CLINIC/HOSPITAL…….<br>COMMUNITY HEALTH VOLUNTEER (ASHA/ASHA PLUS)………<br>FRIENDS/FAMILY…………………………<br>WORKPLACE……………………………..<br>DRAMA GROUPS………………………..<br>POSTERS/BILLBOARDS………………<br>ON TV……………………………………<br>ON THE RADIO…………………………..<br>IN THE NEWSPAPER……………………<br>DAMAN CAMPS_______________<br><br>DON'T KNOW……………………………	All possible numbers to be given rank as the answer(s) received	There will be 5 rank variables with 9 possible values for each of them.<br><br>For rest refer question seven
19	HOW LONG AGO DID YOU SEE OR HEAR THESE MESSAGES?	MONTHS ☐☐		
20	WHAT TYPE OF MALARIA MESSAGES/INFORMATION DID YOU SEE OR HEAR?<br>MULTIPLE RESPONSES POSSIBLE<br><br>PROBE ONCE (ANYTHING ELSE?)	MALARIA IS DANGEROUS……………<br>MOSQUITOES SPREAD MALARIA……<br>SLEEPING UNDER MOSQUITO NET IMPORTANT……………………………<br>SEEK TREATMENT FOR FEVER…….<br>IMPORTANCE OF HOUSE SPRAYING…<br>ENVIRONMENTAL SANITATION ACTIVITIES………………………………<br>DON'T KNOW……………………………	All possible numbers to be given rank as the answer(s) received	There will be 5 rank variables with 6 possible values for each of them.<br><br>For rest refer question seven
21	WHAT INSTANT ACTION DO YOU NEED TO TAKE IF SOMEONE SUFFERS FROM FEVER IN YOUR HOUSE?<br><br>(ONE CORRECT RESPONSE)	DO NOT NEED ANY ACTION………..1<br>VISIT ASHA / MALARIA VOLUNTEER……………..……………2<br>VISIT TO SC/MPHW…………………..3<br>VISIT TO PHC/CHC……………………4<br>SDH/DHH/ MEICAL COLLEGE……….5<br>PRIVATE DRUG SELLING OUTLETS………………………………..6<br>PRIVATE HOSPITALS…………………7<br>INFORMAL PROVIDERS……………...8<br>DON'T KNOW……………………………9		
22	HOW LONG AGO DID MALARIA MOST RECENTLY OCCUR IN YOUR HOUSEHOLD?<br>If During The Same Month Record '0'	RECORD IN MONTHS<br>☐	**IF "DIDN'T OCCUR"** THEN WRITE 999.<br>**IF "DON'T KNOW"** THEN WRITE 998	
23	IS THERE A COMMUNITY HEALTH VOLUNTEER (ASHA/ MALARIA VOLUNTEER) IN YOUR VILLAGE?	YES...1<br>NO...0<br>DON'T KNOW………………………………98	IF 'DON'T KNOW' OR 'NO' SKIP 24,25,28	
24	IF YES, HAVE YOU EVER CONSULTED HER WHEN YOU OR A FAMILY MEMBER HAD A FEVER?	YES...1<br>NO...0<br>DON'T KNOW………………………………98	IF 'DON'T KNOW' OR 'NO' SKIP 25	
25	IF YES, HOW LONG AGO?	LESS THAN 3 MONTHS …………………1<br>3-6 MONTHS AGO………………………..2<br>MORE THAN 6 MONTHS…………..……3<br>DON'T KNOW ……………………………98		

26	HOW LONG DOES IT TAKE TO **TRAVEL** TO THE NEAREST GOVERNMENT HEALTH FACILITY	RECORD IN MINUTES<br>☐ ☐ ☐		
27	HOW LONG DOES IT TAKE TO **TRAVEL** TO THE CLOSEST PRIVATE CLINIC / DRUG SHOP/INFORMAL CLINIC?	RECORD IN MINUTES<br>☐ ☐ ☐		
28	HOW LONG DOES IT TAKE TO **WALK** TO THE ASHA/ MALARIA VOLUNTEER?	RECORD IN MINUTES<br>☐ ☐ ☐		

SECTION 4

VECTOR CONTROL

NO.	QUESTIONS AND FILTERS	CODING CATEGORIES			Instructions data collection	Instructions data entry
29	At any time in the past 6 months, has anyone sprayed the interior walls of your dwelling against mosquitoes?	YES..1<br>NO..0<br>DON'T KNOW...98			If 'NO' OR 'DON'T KNOW' then skip 30	
30	How many months ago was the house sprayed?<br>IF LESS THAN ONE MONTH, RECORD '0' MONTHS AGO.	MONTHS AGO....................... ☐				
31	Does your household have any mosquito nets?	YES 1<br>NO 0			If 'NO' then skip 32 to 38	
32	How many mosquito nets does your household have?	Total NUMBER OF NETS ☐☐				
33	How many nets are?	Supplied by any government body				
		Size	Single	a.		
			Double	b.		
		Self-purchased ever treated				
		Size	Single	a.		
			Double	b.		
		Self-purchased never treated				
		Size	Single	a.		
			Double	b.		
34.	If net were distributed in boarding school, did children bring it back during holidays?	YES..1<br>NO..0<br>NO CHILD IN SCHOOL……………………2				
35	How long ago did you obtain the newest net?	MONTHS AGO, ☐☐				

36	Where did you obtain the newest net?	GOVERNMENT CLINIC/HOSPITAL	1		
		VILLAGE HEALTH COMMITTEE	2		
		ASHA	3		
		RETAIL SHOP	4		
		WORKPLACE	5		
		DON'T KNOW	98		
37	A) How many pre- treated nets were received in the last 2 years?	Number of nets ☐ ☐			
38	A. How many members of your household (including yourself) slept under a mosquito net last night?	Number of persons ☐ ☐			Should not be more than persons in the household. See page 2
	B. If not, why?	Insufficient Net…………………1<br>Weather………………………….2<br>Unwillingness…………………….3<br>Slept outside………………………4			

SECTION 5

NO.	QUESTIONS AND FILTERS	CODING CATEGORIES		Instructions data collection	Instructions data entry
39	Has anyone in the family been ill with a fever at any time in the last 2 weeks?	YES	1	If 'NO' then go to section 6	
		NO	0		
40	If YES, how many people	☐☐			Should not be more than persons in the household. See page 2

SECTION 6
PREGNANT AND LACTATING MOTHER

<table>
<tr><td>Sl no</td><td>QUESTIONS AND FILTERS</td><td colspan="5">CODING CATEGORIES</td><td>Instructions data collection</td></tr>
<tr><td>41</td><td>Age at marriage (Record in years)</td><td colspan="5"></td><td></td></tr>
<tr><td>42</td><td>Number of Pregnancies identified including the present one</td><td colspan="5"></td><td></td></tr>
<tr><td rowspan="5">43</td><td rowspan="5">Detail of previous pregnancy (s)</td><td>S. No</td><td>Age At pregnancy (Record in years)</td><td>Outcome</td><td>Birth weight (in Kg)</td><td>Hb at child birth (In g/dl)</td><td rowspan="5">Start from most distant pregnancy</td></tr>
<tr><td>1</td><td></td><td>1.Live birth
2.Still birth
3.Medical Termination
4.Spontaneous Abortion</td><td></td><td></td></tr>
<tr><td>2</td><td></td><td>1.Live birth
2.Still birth
3.Medical Termination
4.Spontaneous Abortion</td><td></td><td></td></tr>
<tr><td>3</td><td></td><td>1.Live birth
2.Still birth
3.Medical Termination
4.Spontaneous Abortion</td><td></td><td></td></tr>
<tr><td>4</td><td></td><td>1.Live birth
2.Still birth
3.Medical Termination
4.Spontaneous Abortion</td><td></td><td></td></tr>
<tr><td rowspan="3">44</td><td rowspan="3">Current Pregnancy history</td><td colspan="2">Lactating mother</td><td>Date of delivery</td><td colspan="2"></td><td rowspan="3"></td></tr>
<tr><td colspan="2" rowspan="2">Pregnant Mother</td><td>Expected date of delivery</td><td colspan="2"></td></tr>
<tr><td>Date of last menstruation</td><td colspan="2"></td></tr>
<tr><td>45</td><td>Age at first childbirth (record in Years)</td><td colspan="5"></td><td>Enter data both in case of live birth and still birth</td></tr>
<tr><td>46</td><td>History of diseases suffered in the past</td><td colspan="5"></td><td></td></tr>
</table>

Follow up of the current pregnancy:

47	Outcome of pregnancy, including the current one:	S. No	Age	Live birth	Still birth	Medical Termination Of Pregnancy	Spontaneous Abortion	Birth weight	Hb of mother at child birth	Instructions data collection
		1.								In case of twins, fill up both the rows.
		2.								

48. Contact details for follow up

Name	Phone Number
HEAD OF THE HOUSE	
ASHA	
ANGANWADI	
