# Supplementary material for: Assessment of effectiveness of DAMaN: A malaria intervention program initiated by Government of Odisha, India
Source: PLoS One. 2020 Sep 8;15(9):e0238323. doi: 10.1371/journal.pone.0238323 (PMC7478908; doi:10.1371/journal.pone.0238323)
Supplement: Attachment — Submitted filename: Response to Reviewers.docx [file pone.0238323.s002.docx]

# Response to reviewers

## Response to points raised by academic editor

1. Please ensure that your manuscript meets PLOS ONE's style requirements, including those for file naming. The PLOS ONE style templates can be found at

<https://journals.plos.org/plosone/s/file?id=wjVg/PLOSOne_formatting_sample_main_body.pdf> and

<https://journals.plos.org/plosone/s/file?id=ba62/PLOSOne_formatting_sample_title_authors_affiliations.pdf>

Answer1: The manuscript submitted, meets the PLOS ONE’s style requirements. The files have been named in the prescribed style as well.

2.  Please include additional information regarding the survey or questionnaire used in the study and ensure that you have provided sufficient details that others could replicate the analyses. For instance, if you developed a questionnaire as part of this study and it is not under a copyright more restrictive than CC-BY, please include a copy, in both the original language and English, as Supporting Information."

Answer 2: The questionnaire has been attached as “supportive information”.

3. In your Methods section, please provide additional information about the participant recruitment method and the demographic details of your participants. Please ensure you have provided sufficient details to replicate the analyses such as: a) the recruitment date range (month and year), b) a description of any inclusion/exclusion criteria applied to participant recruitment, c) descriptions of where participants will be recruited and where the research will take place.

Answer 3:

a) Each round of survey will be conducted within August to November, which synchronizes with the period for DAMaN implementation

b) The inclusion/exclusion criteria have been defined and necessary edits made in the text.

c) With regards to the location of the research we will select six districts of the Indian state of Odisha which are clearly mentioned in Figure 2 (currently modified as per the suggestions of 1^st^ Reviewer). These will be selected in due course of time.

The above statements have been included in the mansucript

Moreover, in ethics statement in the manuscript and in the online submission form, please provide additional information about the patient records (DAMaN project data) that will be used in the retrospective part of study. Specifically, please ensure that you have discussed whether all data will be fully anonymized before you accessed them and/or whether the IRB or ethics committee waived the requirement for informed consent. If patients provided informed written consent to have data from their medical records used in research, please include this information.'

DAMaN project data will be extracted in aggregate form and no individual-level data will be retrieved or used, therefore there is no need for de-identification of DAMaN project data. There is also no plan to access patient medical records. This has been included in the manuscript in the relevant section. However, for household survey we have included in the manuscript the standard statement for acquiring consent and deidentifying raw data

4. PLOS ONE does not permit references to unpublished data; therefore, we request that you either include the referenced data or remove the instances of "data not shown," "unpublished results," or similar.

 Answer 4: We have cited a website supporting this statement and removed the “unpublished results”

5. We suggest you thoroughly copyedit your manuscript for language usage, spelling, and grammar. If you do not know anyone who can help you do this, you may wish to consider employing a professional scientific editing service.  

Whilst you may use any professional scientific editing service of your choice, PLOS has partnered with both American Journal Experts (AJE) and Editage to provide discounted services to PLOS authors. Both organizations have experience helping authors meet PLOS guidelines and can provide language editing, translation, manuscript formatting, and figure formatting to ensure your manuscript meets our submission guidelines. To take advantage of our partnership with AJE, visit the AJE website (http://learn.aje.com/plos/) for a 15% discount off AJE services. To take advantage of our partnership with Editage, visit the Editage website ([www.editage.com](http://www.editage.com/)) and enter referral code PLOSEDIT for a 15% discount off Editage services.  If the PLOS editorial team finds any language issues in text that either AJE or Editage has edited, the service provider will re-edit the text for free.

Upon resubmission, please provide the following:

a) The name of the colleague or the details of the professional service that edited your manuscript

b) A copy of your manuscript showing your changes by either highlighting them or using track changes (uploaded as a *supporting information* file)

c) A clean copy of the edited manuscript (uploaded as the new *manuscript* file)

 Answer 5: Our colleague, Dr. Ambarish Dutta, has done thorough copyediting of the manuscript for language usage, spelling and grammar.

6. We note that you have stated that you will provide repository information for your data at acceptance. Should your manuscript be accepted for publication, we will hold it until you provide the relevant accession numbers or DOIs necessary to access your data. If you wish to make changes to your Data Availability statement, please describe these changes in your cover letter and we will update your Data Availability statement to reflect the information you provide.

Answer6: We have made changes to our Data Availability statement, which has been described in the cover letter also

## Comments from 1st Reviewer

Figure 2 suggests that two districts are already selected from each of the three strata. Selection of the study districts in each stratum should be unbiased for which, the districts in each stratum may be listed from higher to lower API and then divided further in two subgroups of almost equal number of districts, one districts from upper half and one from the lower half in each zone may be selected. Sample size should be calculated based on lowest prevalence. This sample size may be applied to higher prevalence district in each of the zones for uniformity. Since prevalence of malaria has shown significant reduction in the years 2018 and 2019 in Odisha, sample size calculation should be based on current prevalence/incidence for representativeness and not based on 2016 data.

Answer1: “Selection of 2 DAMaN- implementing districts from each region of Odisha- Northern, Central, Southern; Six districts in total. Within each region, the first district will be selected from those having above median API and the second to be selected from below median API".

This above text within quotes will be now included in Figure 2 that explains the sampling method and design. We are grateful to the reviewer for suggesting an extra layer of stratification (based on API) to make selection of districts really unbiased, hence, the selection is to be carried out now as per the newly suggested method.

However, the sample size has been calculated for all the six districts together and not for individual district as the study objective is to compute pooled estimates mainly and not at district-wise estimates. Moreover, the DAMaN-implementing blocks and Sub-centres have similar transmission intensity so district or sub-district-wise sample size calculation for lower units may not be needed based on variances in incidence. In this context the rarest prevalence that is parasitaemia among pregnant/lactating women had been chosen as the basis for calculating sample size and it is assumed that this prevalence will be roughly similar in DAMaN-implementing pockets of the state.

Additionally, the malaria prevalence data is not routinely collected by the malaria programme so cannot be the basis of sample size calculation. Consequently, the malaria case detection (monthly/yearly) rate (also known as monthly/yearly parasite incidence) which is a proxy for incidence of malaria has formed the basis for selection hotspots by DAMaN, and the year chosen by DAMaN planners was 2016. Therefore, the DAMaN Assessment exercise has also decided to select 2016 as the basis year for sampling. We would like to adhere to this methodology and not select the years 2018 and 2019 as basis years for sampling, during which period there was decline in malaria cases across the state.

2. Introduction: Authors have stated ‘This initiative saw huge investment of public fund from the state government.’ How much per capita was invested, may be mentioned?

Answer 2: Agreed, the exact statement of expenditure has been mentioned in the manuscript

3. “At least 1 ml of intra- venous blood specimen will be collected for malaria and haemoglobin analysis and anthropometric measurements will be recorded from all the members of the sampled household present at the time of interview.”

Considering only two years of implementation of DAMaN, what is the rationale of taking anthropometric measurements? What difference DAMaN would have made in a short span to these features, is not understood? Hence this objective may be dropped and focus should be primarily on the epidemiological impact.

Answer 3:

We would like to include this component for the following reasons

- This anthropometric data may work as a baseline data for future impact evaluation
- We can compare with state anthropometric averages generated by DHS data and understand the situation in DAMaN pockets

4. What definition of an asymptomatic case will be used? Will temperature be taken at the time of house visit?

Answer 4: Self reported data of history of fever in the family in last two weeks before the date of survey will be collected in-order to understand the prevalence of symptomatic malaria. Temperature recording will not be carried out during the survey.

5. Where is P. malariae in the whole scheme? This species has been reported from Odisha with decent prevalence in some districts as in previous studies. [The prevalence of P. malariae in Odisha, India by Pati et. al., Tropical Biomedicine 34(3): 607–614 (2017)]

Answer 5: As mentioned in the manuscript (Page 16), we would assess the prevalence of *P*. *malariae* in this study*. The* diagnosis will be done by using species specific Polymerase Chain Reaction (PCR) as described in the methodology.

6. The rationale for using three diagnostic methods may be clearly stated in view of Pfhrp-2 gene deletion which ranges from 5-10%; e.g. what is missed by RDT could be captured by microscopy and the PCR will capture low/sub-microscopic parasitaemia which the above two methods may miss detection.

Answer 6: Agreed to the comment. As suggested the rational behind using three diagnostic methods, namely Rapid Diagnostic Test (RDT), blood smear microscopy, polymerase chain reaction (PCR) will be used to find the prevelance of malarial parasite in the blood specimen collected. This modus-operandi will ensure an exhaustive investigation for detecting parasitaemia. The Histidine-Rich Protein 2 (PfHRP2) gene deletion in *Plasmodium falciparum* leads to no-detection in RDT, however Pf prevalence in those cases can be captured using microscopy and PCR. The PCR method will also be helpful in detecting parasite at sub-microscopic level, which usually gets missed out by RDT and Microscopy. The same reason three different methods have been incorporated in the text, as suggested.

7. On page 15, objective (d), Remove `prevalence’ to assess the outcome of maternal and child health.

Answer 7: Done. “Prevalence” removed.

8. On page 15, explain outcome assessment from MSAT? (Use Primary data/Generate data)

Answer8: Coverage of MSAT, a component of DAMaN will be estimated along with MSAT’s test positivity rate, proportion of asymptomatics among positives etc. This will be done from DAMaN project data that is secondary data.

9. On page 16, Aim 3: Explain about stratification in detail in methodology.

Answer 9: Sub-centres will be stratified into tertiles based on their DAMaN MSAT coverage and then interrupted time series analysis and difference-in-difference analyses will be carried out in these three strata based on the hypothesis that greater coverage of MSAT will lead to greater decline of malaria. Now expanded in manuscript.

10. On page 17, Sample size: Based on the calculated sample size, you should include 2228 household with pregnant women not household only.

Answer 10: Agreed and that will be done

11. On page 22, Statistical analysis: Anaemia should be defined separately for pregnant women, children and general population in a tabular form. It is not clear.

Answer 11: Thanks again for this very valuable suggestion. Done in the manuscript

12. Authors will need to calculate sample size separately for LLIN usage (No. of Household) and child health outcome (<5 years)

Answer 12: LLIN penetration and usage are reasonably high in these DAMaN areas from current anecdotal information. The current sample will suffice to estimate the LLIN-related proportions with enough precision.

The same logic applies for under-5 children as this large sample size is adequate enough to estimate precise childhood health outcome given further that these health outcomes (such as undernutrition and anaemia) are not at all rare, rather rampant in these hotspots. Moreover, almost every household in these areas will have one under-5 given the current fertility pattern of this population. So, the current sampled household will suffice all the purposes.

## Review from 2nd reviewer

In the main objectives of the DAMaN Assessment the authors stated that they will estimate the mass screening and treatment (MSAT), vector control measures (LLIN and/or IRS) and community mobilization and participation. However, no detailed information was presented on how variables related to vector control measures (LLIN usage, for example) and community mobilization and participation will be measured to be included in the difference analysis models.

Answer: We are extremely grateful to the kind and encouraging words of 2^nd^ reviewer.

In answer to the query these indicators that were mentioned will be estimated using descriptive statistics initially. However, there variations may be modelled against probable explanatory variables, post hoc, if any interesting findings emerge from the descriptive tables.
